# Resilient heritage language maintenance: the interplay of family, culture, and pragmatic choices

**DOI:** 10.3389/fpsyg.2025.1550704

**Published:** 2025-04-10

**Authors:** Orsolya Bilgory-Fazakas, Sharon Armon-Lotem

**Affiliations:** ^1^Department of English Literature and Linguistics, Bar Ilan University, Ramat Gan, Israel; ^2^The Gonda Multidisciplinary Brain Research Center, Bar Ilan University, Ramat Gan, Israel

**Keywords:** multilingualism, heritage language, language transmission, language maintenance, identity, parental reports

## Abstract

**Introduction:**

Globalization and transnational mobility have contributed to linguistic and cultural diversity. Yet small migrant communities trying to preserve their heritage language (HL) face challenges. This study investigates the intersection of family language policies (FLPs), migration, and multilingualism within the Hungarian-speaking immigrant community in Israel, focusing on the social and affective dimensions of HL transmission. Grounded in the FLP framework, it explores how cultural heritage, parental attitudes, and pragmatic considerations shape HL transmission and maintenance, particularly within transnational families, where maintaining ties with extended family often serves as a key motivation.

**Methods:**

The study involved 51 Hungarian-speaking adults who immigrated to Israel post-2000, with at least one child under 18. Participants were functional multilinguals who used Hungarian (HL), Hebrew (societal language, SL), and English daily. An online questionnaire assessed participants’ biographical information, language use, attitudes toward HL maintenance, and code-switching. Self-rated language fluency was measured using the CEFR scale, and data were analysed using Principal Component Analysis (PCA) and multilinear regression to identify predictors of HL transmission and maintenance. Thematic analyses (TA) were used to identify and report themes within the qualitative data.

**Results:**

Findings revealed that most families adopted bilingual FLPs, balancing HL and societal language (SL) use. PCA identified two significant components: cultural heritage (including parental emphasis on HL literacy, cultural practices, and linguistic immersion) and communication in HL (focused on child-directed HL use). Parental attitudes towards code-switching negatively correlated with HL use, while efforts to transmit cultural heritage positively predicted successful HL maintenance. Multilinear regression analysis identified cultural heritage transmission and attitudes toward code-switching as significant predictors of HL maintenance. Grandparents played a central role in encouraging HL transmission and maintenance, with strong correlations observed between parental efforts and children’s ability to communicate with extended family.

**Conclusion:**

This study examines how Hungarian-speaking immigrant families in Israel, a small and underrepresented community, maintain their HL. The findings suggest that balanced exposure to both HL and the SL supports HL sustainability, with intergenerational ties playing a key role. However, the study’s scope is limited to highly educated, mid-high SES families, so results may not apply to the entire community.

## 1 Introduction

Multilingualism defines the twenty-first century while presenting opportunities and challenges, particularly to small immigrant communities striving to sustain their heritage languages (HL). The study aims to enhance our understanding of HL transmission, development and maintenance by examining how family language policy (FLP) and cultural identity interact in these dynamic multilingual settings. Within these communities, members of transnational families live in different countries but maintain family connections with each other (Bryceson and Vuorela, 2002). In this context, older members of the extended family are often also the reason to prioritize transmitting and maintaining a HL as a part of their cultural identity and as a connection to their families, as they do not learn the SL (Braun, 2012). On the other hand, language practices and attitudes shift within families and generations. Sometimes, the paths of older and younger generations take different directions, moving from preservation to assimilation or vice versa. The linguistic landscape of Israel, a nation known for its rich immigrant cultures with larger and smaller migrant communities, is diverse. Despite Hebrew being the official language (Spolsky and Shohamy, 1999), Israel remains a multicultural and multilingual society. This language diversity results from Jews who immigrated to Israel and brought their community languages with them. While acquiring Modern Hebrew, they also transmitted and maintained them as HLs. It is also evident in the significant proportion of the population that speaks HLs, including Russian, English, French, Spanish, and Hungarian, among others (Altman et al., 2013; Ben-Rafael and Ben-Rafael, 2018; Meir et al., 2021). According to the 2011 Central Bureau of Statistics (hereafter, CBS; CBS, 2011) Lifetime Learning and Usage of Languages Survey, 49% of the population consider Hebrew their native language, followed by Arabic, 18%, and Russian, 15%. The remaining 18% of the population listed Yiddish, French, English, Spanish and others as their native language (Meir et al., 2021). The country also ranks fourth in the OECD in terms of the number of immigrants in its population (source: OECD/European Commission, 2023a).

The Hungarian-speaking population presents a particularly intriguing case among these communities, as this community has seen significant changes over the decades. Studies describing Hungarian communities abroad and in the homeland highlight their non-cohesive characteristics. Therefore, different waves of Hungarian migrants show divergent traits (Fenyvesi, 1995–1996, 2005; Bartha, 1995; Hatoss, 2006, 2016; Kontra, 1990, Szilágyi and Szécsi, 2020). The Hungarian-speaking community had 13,616 members in Israel in 1948, and 24,143 people arrived from Hungary from 1948 to 1960 (CBS, 2010). However, these numbers are only estimated because these people immigrated from Hungary and the neighboring countries. Overall, the Hungarian-speaking community is ageing; those who came in 1940–60 acquired Modern Hebrew and assimilated into Israeli society, often at the cost of transmitting Hungarian to the second generations, resulting in notable language attrition among its members (Ben-Rafael, 1994; Roseunhouse, 2012). According to Ben-Rafael’s (1994) study, although the Hungarian language has high ethnic significance, the younger generation shows significant language attrition. After the fall of communism in 1989 and until recently (2016), the number of migrants from Hungary hardly exceeded 3,800. There were two historical events in the aforementioned period when their number slightly increased: the first was right after the fall of the communist regime due to freedom of movement and the restoration of diplomatic relations between the two countries. The next historical event was the global economic crisis in 2008 when the number of immigrants grew again. Yet, the fall of communism and the subsequent periods of increased migration have introduced new dynamics to this community as the earlier Hungarian-speaking immigrants experienced significant language attrition while assimilating into the Israeli society, often prioritizing the SL over the HL. However, the newer waves of migrants brought with them a different linguistic and cultural outlook influenced by European values of multilingualism.

The Hungarian-speaking immigrant community in Israel stands in the focus of this paper. This community has undergone significant demographic and linguistic changes shaped by different waves of migration. The study’s participants are part of this last wave of Hungarian-speaking immigrants. These participants grew up in Hungary after the fall of communism and experienced socialization in a country that became a member of the European Union (EU). In this environment, multilingualism was widely encouraged, both culturally and institutionally. For example, the EU requires proficiency in multiple languages for higher education degrees and professional careers (Multilingualism, 2009). This emphasis on multilingual competency is reflected in the participants’ biographic data, as most of them have conversational fluency in English. Broader societal views also shape their attitudes toward language learning: according to the Eurobarometer (2023) survey, 85% of Hungarians believe that everyone should speak at least one language other than Hungarian and 68% more than one additional language. Furthermore, 81% believe that regional and minority languages should be protected, reinforcing the idea that HL maintenance is a valued cultural goal in their home country (source: Eurobarometer on Hungary).

The study explores FLP used by the Hungarian-speaking community members in Israel and its impact on the transmission and maintenance of Hungarian as a heritage language within this community, where Hebrew is the societal language. Utilizing the framework of FLP (Spolsky, 2004; Curdt-Christiansen, 2014), this research aims to provide insights into how small migrant communities navigate the challenges of preserving their linguistic heritage in an environment where the SL is predominantly used. It seeks to understand the interplay between cultural heritage and pragmatic considerations in HL maintenance. It hypothesizes that parental attitudes, FLP, and identity considerations significantly influence the transmission and maintenance of Hungarian within the family. The study predicts that a strong commitment to cultural heritage will enhance HL preservation. Additionally, it investigates the potential negative impact of attitudes towards code-switching on HL maintenance, suggesting that discouraging code-switching may promote more consistent use of the HL. Ultimately, the research aims to contribute to the broader understanding of the multilingual and multicultural dynamics between assimilation and preservation that enable transnational families within small immigrant communities to sustain their linguistic and cultural identities in a globalized world.

### 1.1 Transnational identity and families

International mobility rooted in globalization, information and communication technology yields linguistic and cultural diversity in the identity of immigrants, resulting in transnational identity. The concept of ‘transnational identity’ is used in several studies referring to a certain lifestyle of immigrants who maintain their ties with their home country while building up new roles, beliefs and ideas within the host country (Esteban-Guitart et al., 2013; Portes, 1997, 2001; Suarez-Orozco and Suarez-Orozco, 2001). This identity formation is particularly influenced by family structures, as immigrant families play a key role in how individuals balance their cultural heritage and integration into the host society. Suarez-Orozco and Suarez-Orozco (2001) studied the adaptations of immigrant children and youth, and according to their results, they proposed three main ways immigrants construct their identity: ethnic flight, active opposition, and biculturalism. In ethnic flight, immigrants abandon their HL and culture to fully adopt the dominant culture fully, often losing ties with their original family traditions and language. On the other hand, active opposition involves rejecting the dominant culture after feeling rejected by it, often leading to forming separate identity linked exclusively to their heritage country. Yet, the most common approach, as found by the authors, is biculturalism, where immigrant children blend their heritage identity and family traditions with the new culture, acquiring multicultural skills and maintaining ties with both cultures. It allows individuals to preserve emotional connections with their culture of origin while gaining the necessary skills to succeed in the dominant culture. Suarez-Orozco and Suarez-Orozco (2001) argue that the bicultural approach is the most adaptive, where ongoing interactions with relatives in both the heritage and host countries shape identity. In this context, family structure plays a crucial role in identity formation. While some communities emphasize the nuclear family of parents and children, others maintain an extended family system, including grandparents (Poeze and Mazzucato, 2014). Bryceson and Vuorela (2002) define transnational families whose members are motivated to maintain close and active family relations regardless of geographic distance. They live apart for extended periods or permanently yet maintain a sense of shared well-being, unity, and familial connection across national boundaries, thus the relationships among family members are maintained through digital technology and air travel. This enables the family members to engage in the social and cultural domains, where a hybrid transnational identity and language practice can easily coexist.

The linguistic choices within the family unit—whether to emphasize the HL or prioritize the SL—reflect deeper identity negotiations and reveal the complex interplay between assimilation and cultural preservation. These families are dynamic and rooted in the variations of family circumstances, and their HL use is in constant negotiation. The negotiation of HL within transnational families illustrates how language is not just a tool for communication but a fundamental element of identity formation and intergenerational connection (Bryceson and Vuorela, 2002; Tannenbaum and Howie, 2002; Curdt-Christiansen, 2009; Mazzucato, 2014).

### 1.2 Family language policy

The success of transmitting HL in multilingual transnational families is also related to parental attitudes, beliefs, and efforts towards using HL, all of which form the family language policy (FLP). Therefore, FLP is responsible for the HL environment and refers to the overall linguistic setting within the family that provides the maintenance and transmission of the HL. Spolsky’s (2004) model is often used as a theoretical framework to describe the three domains that FLP encompasses: language ideology (e.g., parental beliefs), language practices (e.g., parental language input and linguistic behavior), and language management (e.g., direct attempts to affect the language practice). These domains are strongly interrelated, as the language ideology can govern the actual practices but can simultaneously be influenced by them (Altman et al., 2013).

The theory has been expanded by King and Fogle (2008, p. 4), who argue that FLP may influence children’s cognitive development and contribute to their performance in institutional settings and can have an emotional impact on them (King and Lanza, 2019, p. 15). Curdt-Christiansen (2014) also broadened the theory by highlighting values and perceptions tied to specific languages and deciding what languages are practiced, encouraged, avoided, or abandoned highlighting language ideology as a central factor of the theory. Therefore, the family’s decision-making processes depend on parental beliefs and goals for their children’s linguistic development. They are also influenced by parental education, the family’s socioeconomic status (SES), and prior language-learning experience. Several studies emphasize the importance of close family members who remained in the home country and their role in the HL’s vitality (Guardado and Becker, 2014; Tannenbaum, 2005).

Parental attitudes towards multilingualism significantly impact HL maintenance. Positive attitudes foster an environment where HL is valued and actively used (Curdt-Christiansen, 2009; De Houwer, 1999; King and Fogle, 2008; Schwartz, 2008). Research by De Houwer (1999) and King and Fogle (2008) suggests that parents who view bilingualism as an asset are more likely to implement effective strategies for HL maintenance, such as consistent use of HL at home, enrolling children in HL classes, and creating opportunities for HL exposure.

As home language practices can promote or deter HL transmission and maintenance in this context, parents who intend to maintain HL use various strategies to achieve their goal of raising bilingual children (Lanza, 1997; King and Fogle, 2006; Curdt-Christiansen, 2014). Therefore, it is essential to understand whether families choose monolingual or bilingual FLP. Parents may speak different languages at home (e.g., the One Parent One Language approach—OPOL), use only HL at home, or use two languages within the household. It has been shown that assuming that children who are born in bilingual families acquire the languages naturally does not result in active bilingualism, and parental language practices and attitudes are critical factors in bilingual development and shape children’s HL outcomes (De Houwer, 2007). Parents who view bilingualism as a goal adopt strategies that foster HL use. Lanza (1997) identifies five parental interaction strategies (minimal grasp., expressed guess, repetition, move-on and code-switching), that influence how children understand language boundaries. Literacy support and frequent travel to HL-speaking regions strengthen HL maintenance (King and Fogle, 2006). Curdt-Christiansen (2014) emphasizes that FLP is not just a private family decision but is shaped by sociopolitical realities. While positive parental attitudes and consistent HL use at home are essential for HL maintenance, external factors, such as educational policies, economic pressures, societal attitudes and institutional support or lack of them can either reinforce or undermine HL transmission and maintenance.

### 1.3 Heritage languages and their speakers

HL research has recently gained importance due to the public discourse around migration. HLs are minority languages inherited within the family and used in the home domain, which may be acquired simultaneously or sequentially to the SL (Valdés, 2000). Heritage speakers (HS) are second-generation immigrants exposed to a variety of HL in natural situations at home during childhood. They maintain the HL to various degrees and often exhibit uneven linguistic outcomes, for example, different levels of proficiency in various language skills (speaking, listening, reading, and writing) due to factors like exposure and formal education (Montrul, 2008). They experience reduced language input in their HL because it is used in the family domain in contrast to the SL used in other domains, including access to institutional support in the SL as schooling occurs in that language. Thus, HSs typically have more vital skills in listening and speaking than reading and writing. Due to the absence of or limited schooling in the HL, speech perception is the most developed competence based on self-reports (Carreira and Kagan, 2011; Montrul and Ionin, 2012). The acquisition of literacy skills and formal registers of the HL depend on the actual FLP practice the parents follow (Kupisch, 2013; Brehmer et al., 2020).

Research indicates that community size, density, and geographic concentration can influence HL maintenance. Pauwels (2016) highlights that larger HL-speaking communities can provide greater exposure through agencies, institutional support, and social reinforcement. Access to HL schools, media, and cultural spaces helps sustain the HL across generations. These factors also contribute to variations in HL proficiency, with larger communities showing higher levels of bilingual fluency (Gollan et al., 2015). In sum, the success of transmitting the HL is often related to the size of the HL community and the density of HSs within the area (Meir et al., 2021), the institutional support of the home and host country toward the HLs and the parental desire to maintain family relations with the relatives left in the home country.

### 1.4 Cultural heritage and pragmatic considerations in heritage language maintenance

The interplay between cultural heritage and pragmatic considerations provides a comprehensive framework for understanding HL maintenance. Cultural heritage offers the emotional and identity-driven foundation for language practices, while pragmatic considerations provide practical motives that reinforce daily language use (De Houwer, 2007; Schwartz, 2008; Tannenbaum and Howie, 2002). Parents who strongly identify with their cultural background are more committed to transmitting the HL to their children. Studies have shown that these parents often engage in cultural practices, celebrate cultural festivals, and emphasize the importance of knowing the HL to maintain cultural ties (Guardado and Becker, 2014; Curdt-Christiansen, 2009). This cultural identity motivation can lead to more robust and consistent language policies within the family. Pragmatic reasons, particularly the need for communication with extended family members and educational benefits, also play a crucial role in HL maintenance. Mu and Dooley (2015) highlight the importance of the HL in facilitating communication with family members, strengthening familial bonds and preserving family traditions. A longitudinal study on HL transmission in Australia has further supported this notion. The study highlights the importance of personal factors, such as parental HL use, the physical presence of grandparent(s), and environmental factors, including parental perceptions of educational support and first- and second-generation immigration status, in maintaining an HL (Verdon et al., 2014). When comparing pragmatic considerations with ideological or economic reasons for HL maintenance, recent studies reveal that while all these factors are vital, pragmatic considerations often yield more immediate effects (Ortega, 2019). This multifaceted approach ensures that HLs are preserved not only as cultural assets but also as functional tools for communication and cognitive development. This balanced strategy is essential for the sustainable maintenance of HLs within immigrant families, addressing both the emotional and practical aspects of language use and transmission.

### 1.5 Attitudes towards language use and code-switching

Parental agency significantly influences children’s language use and literacy outcomes, and research has shown that children’s agency in HL use often mirrors their parents’ level of commitment and active involvement in promoting HL (Wei, 2006; King and Fogle, 2008; Shen and Jiang, 2023). Children typically acquire their language(s) based on adults’ input, and language use patterns within the home shape their linguistic development. In multilingual households, using more than one language at home might give rise to a prominent feature of bilingual speech: code-switching (CS). The practice of alternating between two or more languages within a conversation is a common feature among multilingual speakers (Grosjean, 2008; Hoff et al., 2012; Paradis, 2007). However, attitudes towards CS may vary across different social and linguistic contexts.

Several studies have examined parental attitudes towards CS but have not reached common ground. On the one hand, some researchers argue that CS can be a linguistic strategy to enhance communication and language acquisition. It can facilitate language learning by providing contextual clues and aiding in transferring linguistic structures across languages (Mac Swan, 2000; Lee, 2006; Bullock and Toribio, 2009). On the other hand, other studies found that discouraging CS help children differentiate between languages and maintain both languages more effectively (Lanza, 2007; Paradis, 2007). Grosjean’s work (2008) on bilingualism emphasizes that while CS is a natural part of bilingual communication, some communities view it negatively, and it can lead to stricter FLP. In such cases, parents may actively promote HL use while restricting CS and reinforcing linguisticboundaries.

### 1.6 Developmental and environmental factors in the reported parental language use

Developmental and environmental factors including the age of the child, the age of onset of bilingualism, the quantity and quality of the input the child receives in the target languages, the family’s SES, parental education and occupation, birth order, and family size, can affect children’s language development (Paradis, 2010). Most studies that explore second-language acquisition in children focus on examining developmental factors (age or time-related) and their effects on language outcomes at the level of morphosyntax (Chondrogianni and Marinis, 2011; Meir and Armon-Lotem, 2015). However, studies indicate that environmental factors are responsible for more variation in language performance than cognitive factors (Paradis and Jia, 2016). Environmental factors have been identified as related to vocabulary acquisition and general academic success (Oller and Eilers, 2002; Schwartz, 2014). The quality and quantity of oral interactions may differ based on SES—children of high SES receive more qualitative and quantitative adult input than children of low SES in which adult input is reduced (Armon-Lotem et al., 2011). Furthermore, a mother’s self-rated linguistic proficiency effectively predicts a child’s development of the (complex) morphosyntactic features (Chondrogianni and Marinis, 2011; Hoff, 2003). Sociolinguistic factors, such as ethnolinguistic identity, sociopolitical prestige of the HL within the society (Altman et al., 2013; Walters et al., 2014) and parental attitudes impact children’s language-learning outcomes (Armon-Lotem et al., 2015). It is suggested that the density of heritage speakers in the children’s community plays a crucial role in the ethnolinguistic vitality of a particular community, therefore leading to a higher level of proficiency (Gollan et al., 2015; Maneva, 2010).

The objective of the current study is to gain insight into the driving forces of HL transmission and maintenance within a small migrant community, the Hungarian-speaking community in Israel, applying the framework of FLP (Curdt-Christiansen, 2014; Spolsky, 2004) to see whether patterns observed in large migrant communities are also prevalent in small communities. Therefore, the following research questions (RQs) are addressed:

*RQ1.* What types of FLPs are observed among the Hungarian-speaking immigrants in Israel?

*RQ2.* How do personal and environmental identity factors influence heritage and SL acquisition and fluency among the studied community?

*RQ3.* How do parental attitudes, family language policies, and identity considerations shape the transmission and maintenance of Hungarian as HL within immigrant families?

*RQ4.* What are the predictors and determinants of successful HL maintenance, including factors such as length of residence, parental fluency in the SL, and attitudes towards CS?

*RQ5.* What roles do cultural heritage promotion, communication practices, and exposure to the HL play in fostering language transmission and maintenance within immigrant households?

*RQ6.* How do pragmatic considerations, such as intergenerational communication and literacy development, influence parental motivations and efforts towards bilingual education and HL maintenance?

To address these research questions, the study formulates the following hypotheses:

*H1.* The study predicts that different types of FLP are observed among the Hungarian-speaking immigrants in Israel and that families balancing the use of HL and SL at home will be more effective in maintaining HL.

*H2*. Individuals strongly identifying with their cultural heritage are more likely to prioritize HL maintenance and fluency.

*H3*. It is assumed that parental attitudes, FLP, and identity considerations significantly shape immigrant families’ transmission and maintenance of HL.

*H4*. The study also presumes that parental attitudes towards code-switching negatively impact HL maintenance, proposing that discouraging CS in favor of consistent HL use will result in more successful HL preservation. Furthermore, successful HL maintenance is expected to be predicted by factors such as the importance placed on cultural heritage, communication practices, and exposure to HL. While the length of residence in Israel and parental fluency in Hebrew are considered, these factors may not be significant predictors.

*H5*. It is assumed that parents who highly value cultural heritage and maintain a solid Hungarian identity are more likely to engage in HL-promoting practices, such as reading Hungarian stories to their children and ensuring they spend time in Hungary for linguistic immersion.

*H6*. It suggests that parental efforts to foster HL literacy and maintain regular use of the HL within the family are critical predictors of successful transmission. It is hypothesized that parents’ motivations are deeply rooted in the desire to preserve cultural connections and ensure their children can communicate with extended family members, highlighting the practical and cultural dimensions of HL maintenance.

## 2 Materials and methods

### 2.1 Participants

The study was conducted between 2020 and 2021. The participants of the study involved 51 adult members of the Hungarian-speaking community in Israel [47 mothers (92%) and 4 fathers (8%)]. The average age of the participants is 42.93 (SD = 6.88), and they have lived in Israel for 13.74 years (SD = 7.63). The participants are recent immigrants from Hungary who find multilingualism an asset. They all immigrated to Israel after the fall of Communism in Hungary in 1989 and had at least one child under 18 born in Israel. The participants were functional multilinguals, using Hungarian (HL, L1), Hebrew (SL, Heb., L3), and English (Eng., L2) daily. They were recruited for the study through personal connections, word of mouth and the social network sites of an online news site. A 101 questionnaires were returned, but only half of the participants met the inclusionary criteria of having migrated after 2000 and having at least one child under 18 born in Israel who can count as a heritage speaker.

Twenty-nine participants were born in Budapest, Hungary, seventeen in the countryside, and five in neighboring countries (Romania, Serbia and Russia), of whom one was raised in Budapest. The number of children under 18 is 65, and the average age is 7.24 years (SD = 4.96). Their immigration to Israel after 1990 was driven by personal (31), religious (7), economic (5), political (4) or other (4) reasons. Compared to other communities that immigrated after 1990, like the Russian-speaking community was led by economic considerations, and ideological reasons drove the English-speaking. The Hungarian-speaking community was driven mainly by personal reasons (e.g., better career opportunities, trying themselves out in a different cultural setting, etc.) (see Table 1).

**Table 1:** Demographic information of the participants.

Participants				
Number of adult participants	Age of adult participants	Average age of immigration	Number of children	Age of children
51	M = 42.93 years (SD = 6.88 years)	M = 13.74 years (SD = 7.63 years)	65	M = 7.24 years (SD = 4.96 years)

The participants were asked to rate on a 6-point scale their language knowledge in their native language (HL, L1), English (L2) and Hebrew (SL, L3), according to the Common European Framework of Reference for Languages (CEFR) (Table 2), where the A1 level is equal to 1, and the C2 level is equal to 6. The 6-point scale reflects those levels and was used only for self-reporting proficiency in L2 (English) and L3 (Hebrew). All participants marked Hungarian as their first language (L1). They learned English during schooling and used it as a second language (L2) on different levels (25 participants on C1 and C2 levels, 14 on B2 and eight on B1 levels, and 2 on A1 and two on A2 levels). Most participants have conversational fluency in English (94%, M = 3.41, SD = 1.09). Hebrew is their L3. According to the participants’ self-reports, 94% reached conversational fluency (B1 level and above) in Hebrew (M = 3.13, SD = 1.11), of whom 22 are proficient users of Hebrew.

**Table 2:** Language proficiency in L2 and L3.

CEFR level*	L2	L3
C1 + C2	*n* = 25	*n* = 22
B2	*n* = 14	*n* = 19
B1	*n* = 8	*n* = 7
A1 + A2	*n* = 4	*n* = 3

*CEFR, Common European Framework of Reference for Languages.

According to the OECD country report, 26% of the total Israeli population is foreign-born citizens who are, on average, more educated than in other OECD countries. Of these, 46% are highly educated (those individuals who attained tertiary education, OECD, 2023b), compared to 31% in other OECD countries (OECD, 2023b). The participants’ educational background mirrors the OECD report; most (*n* = 40) studied in higher education institutes in Hungary. Many universities are state-owned, and Hungarian citizens can study in a higher educational institute for 10 or 12 semesters in state-funded positions. Therefore, earning an academic degree is highly accessible. Two participants studied in Hungary and Israel, four participants studied in Israel, and three in other countries (Germany, the UK, and the USA). Therefore, the majority of participants are from mid-high SES as they featured various educational and occupational backgrounds (31 positions require higher education degree: doctors, teachers, managers and analysts, designers, photographers; and 20 positions do not, e.g., baker, swimming teacher, tour guide, technician). Many families have grandparents and extended families in Hungary (90%) who would not be able to interact with the children if they did not acquire the HL. Therefore, parental views on the importance of transmitting and maintaining the HL are focused on oral language skills.

### 2.2 Materials and procedure

An anonymous online questionnaire was developed to investigate the community’s language ideologies, practices, and patterns of language management (based on Dörnyei and Csizér, 2002; Lanstyák, 2000). The 5-point Likert scale questionnaire was comprised of separate sections which elicited (1) biographical information (23 items); (2) self-reported first and second language use (44 items); (3) motivation and information towards immigration, second language learning, identity changes (51 items); (4) attitudes toward code-switching (7 items), and (5) attitudes towards the transmission of the HL (17 items) and two open-ended questions.

The demographic information included the age, gender, country of birth, the highest level of education, profession before and after immigration, self-rated language knowledge in the HL, SL and English, languages used in the household, number of years in Israel, access to the Hungarian ethnic community, frequency of contact with other Hungarian families, and opportunities for language use, followed by 51 statements centered around identity, 17 statements centered on the importance of the Hungarian language maintenance, including strategies and difficulties related to HL maintenance. The participants were asked to express their perceptions based on a Likert-type scale ranging from 1 (strongly disagree) to 5 (strongly agree). The two open-ended questions aimed at parental considerations regarding acquiring HL literacy. The online questionnaire and consent letter were distributed through several Facebook groups (Tel-Aviv Parents Support Group, Israeli Hungarians-Izraeli Magyarok-, Hungarian-speaking Israelis-Magyar ajkú izraeliek-, Online meeting forum of Hungarian-speaking parents who live in Israel-Magyar anyanyelvű, and Izraelben élő szülők online találkozóhelye) and online news sites (Új Kelet Online, Izraelinfo) and snowball technic was used too. The study was approved by the Institutional Review Board of XY University, Z.

### 2.3 Data analysis

Quantitative analyses were used to answer the research questions. First, several principal component analyses were carried out to reduce the number of variables represented by the Likert-type items. PCA with Direct Oblimin Rotations was used to reduce dimensionality and determine the variables contributing to parents’ perception of HL transmission and maintenance. As an oblique rotation method, it generates more accurate results than orthogonal rotation methods when there are correlations between the underlying factors (Brown, 2006). PCA was also conducted on the five-item scale to understand participants’ identity regarding their choice of living. The Kaiser–Meyer–Olkin (KMO) measure verified the sampling adequacy for the analysis. Then, using the extracted constructs as dependent variables, multiple linear regression analysis was carried out to discover to what extent these factors contribute to parents’ view on HL maintenance or, more specifically, to find out the extent to which variables identified in the research questions (i.e., family heritage, FLP, environmental or personal factors, attitudes towards code-switching) predicted the specific constructs of parents’ views on HL transmission and maintenance.

Thematic analyses (TA) (Braun and Clarke, 2006) were used to identify and report themes within the qualitative data. An inductive approach was employed for thematic analysis, allowing themes to emerge directly from the data without previously defined categories. The responses were coded and grouped, providing insight into parents’ motivations for HL transmission, such as intergenerational communication, literacy development, and bilingual education.

## 3 Results

### 3.1 Family language policy

**GRAPH 1:**
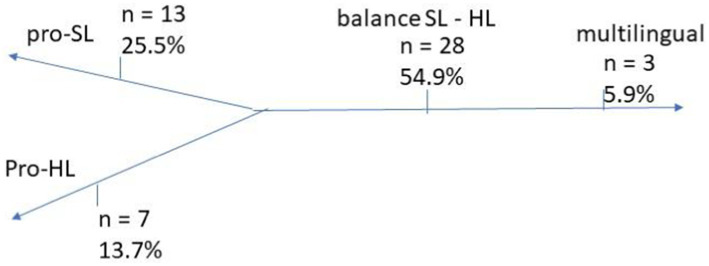
The family language policy and the dominant language spoken at home. The scores are presented as percentages and the number of participants.

This section examines the FLPs observed among Hungarian-speaking immigrants in Israel, addressing RQ1. As the definition of heritage speaker highlights the different ranges of the bilingual continuum, reflecting the enormous variation in HL ability (Polinsky and Montrul, 2013, p. 133), the FLP reported by the study participants is presented in a continuum (Graph 1).

Participants reported their language use within their households: Most (twenty-eight) households use the HL and SL equally, while thirteen use the SL dominantly, seven use the HL dominantly, and only three participants reported using three languages in their households.

### 3.2 Identity factors

The role of personal and environmental identity factors is examined in this section, addressing RQ2. The immigration of most participants was led by personal reasons followed by religious, economic, and political reasons, resulting in transnational and transcultural families in Israel. The KMO = 0.703 (*p* < 0.001) suggests that the sample size is adequate for component analysis. Regarding the identity of the participants, based on the Likert-scale items, two dimensions emerged, 67.518% of the total variation. The first PC, based on *Personal Choice*, comes from items that express their new identity: “I feel I became Israeli.” (item loading is b = 0.953); “I am Israeli.” (b = 0.797); and “I want to become an Israeli.” (b = 0.705). Personal Choice emphasizes newly emerged, conscious identity choices of the participants and accounted for 64.777% of the total variance. The second PC, named *Environmental Reasons*: appear from items that describe the participants’ HL identity, importance of the heritage country and their living arrangements: “I am Hungarian who lives in Israel.” (b = 0.983); “I live in Israel because I want to live here.” (b = 0.710) and “It is important to me to visit and spend time in Hungary.” (b = 0.511).

The second PCA measured participants’ active role in acquiring and fostering the SL. They acquired the SL within 5 years and reached conversational fluency. Based on the PCA, only one component emerged from the Likert-scale items measuring the participants’ self-reports. The KMO = 0.739 (*p* < 0.001) measure confirmed the acceptability of the test. The *SL Fluency* PC emerged with 66.392% of the total variation based on the following items: “I try to use Hebrew in most of the situations.” (b = 0.902); “Speaking Hebrew is important to me.” (b = 0.843); “I speak Hebrew fluently.” (b = 0.757) and “I study Hebrew as much as I can.” (b = 0.748). All items contributed to this component positively correlated with it and related to the values of SL and motivational factors to acquire it.

### 3.3 Elements of heritage language transmission and maintenance

RQ3. Investigates how parental attitudes, FLP, and identity considerations influence the transmission and maintenance of Hungarian as a HL. Those items that measured parental efforts towards HL transmission and maintenance were analysed. The KMO = 0.847 (*p* < 0.001) verifies the sampling adequacy for the study. The result indicates that the two components had an eigenvalue of >1 and explained 77.884% of the variance. The first dimension, the *Cultural Heritage*, is derived from: “It is important to me that my child(ren) get(s) to know the Hungarian culture.” (b = 0.944); “It is important to me to read stories in Hungarian to my child(ren).” (b = 0.868); “It is important to me that my children will acquire literacy skills in Hungarian.” (b = 0.863) and “It is important to me that my child(ren) spend time in Hungary and have the chance for linguistic immersion.” (b = 0.693), highlighting the cultural factors of HL transmission and significance of promoting literacy skills (M = 4.18; SD = 1.12). The following statements: “It is important to me that my child(ren) will know Hungarian. (b = 0.965);” It is important to me that my child(ren) can speak in Hungarian with my family who lives in Hungary.” (b = 0.878); “I am prepared to put my effort in keeping the Hungarian language.” (b = 0.703) and “It is important to me to speak Hungarian while I spend time with my child(ren).” (b = 0.485) elicited the second dimension, *Communication in the HL*, meaning the child-directed speech in the HL. The average mean of 4.55 (SD = 0.844) facilitates the value of HL and parental efforts to transmit and maintain it.

RQ 4. Addresses the predictors of successful HL maintenance, including factors such as length of residence, parental fluency in the SL, and attitudes towards CS. As heritage speakers are exposed to non-monolingual input, questionnaire items measured parental attitudes toward CS. Cronbach alpha has shown good to adequate reliability of the scales regarding an adult (*α* = 0.876) and a child (α = 0.679). Scores over.650 are considered acceptable according to Beaumont (2012) and above 0.60 based on Dörnyei and Dewaele (2023).

**GRAPH 2:**
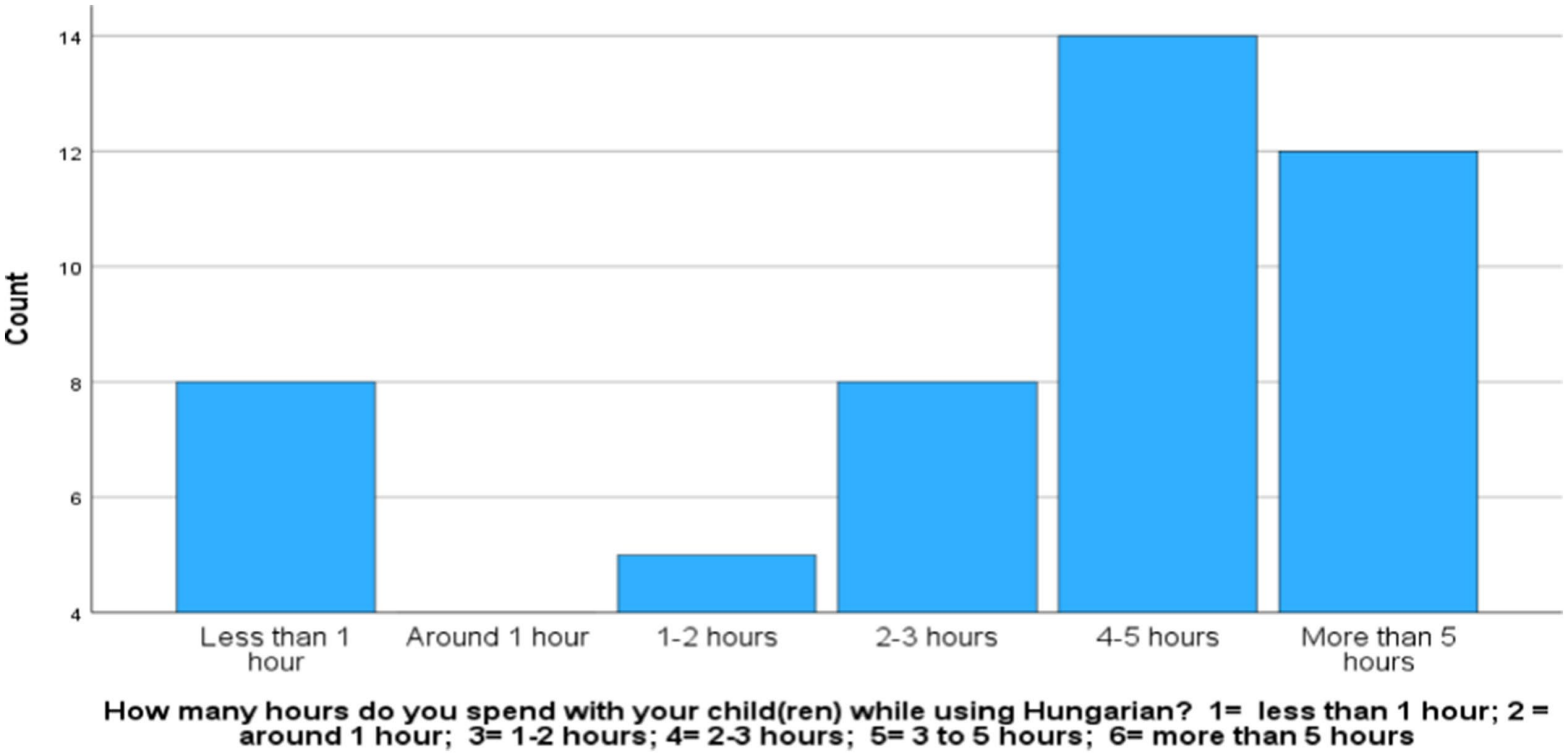
Exposure to HL.

Even though the time spent with the children (Graph 2) does not significantly differ from the daily needed 2–3 h of the children’s awake time (De Houwer, 2007; Hoff et al., 2012) using One Sample Wilcoxon signed test (W = 470, *p* = 0.971), the frequency data shows that 33% of the participants spend less time, which contradicts with the finding of highly rated importance of HL maintenance.

Spearman correlations confirmed a significant association between exposure to HL and HL maintenance (*ρ*r = 0.584, *p* = 0.00001) and cultural heritage transmission (ρ = 0.387, *p* = 0.005). Parents have a negative attitude towards code-switching (ρ = −0.506, *p* < 0.001) in the HL environment. At the same time, parental fluency in the SL does not affect the time parents spend with the children (ρ = 0.077, *p* = 0.599), the HL use (ρ = −0.144, *p* = 0.33) and the attitudes towards code-switching (ρ = −0.075, *p* = 0.652) and the cultural aspects (ρ = −0.128, *p* < 0.387) of HL transmission.

RQ5. Examines the impact of cultural heritage promotion, communication practices, and exposure to the HL. To analyze these factors, the Kruskal Wallis test was used with Mann Whitney *post hoc* analysis to measure the differences between exclusively pro-HL, pro-SL, and balanced SL-HL families in the attitude toward Cultural Heritage, Communication in the HL and parental attitudes towards code-switching (CS Child). A significant difference has been found between the three groups in Cultural Heritage (H = 6.407, *p* = 0.041) and Communication in HL (H = 10.866, *p* = 0.004) but not in CS Child (H = 1.423, *p* = 0.491). *Post hoc* analyses found that the results emerged from the differences between pro-SL and balanced SL-HL families (Cultural Heritage, U = 89, *p* = 0.015; Communication in HL, U = 72, *p* = 0.001). The findings suggest that heritage maintenance is the least important for pro-SL families and the most important for balanced SL-HL families; the pro-HL families are placed between the abovementioned groups.

### 3.4 Predictors of heritage language transmission and maintenance

To explore the factors influencing HL maintenance, this section addresses RQ4 by analyzing key predictors, such as length of residence, parental fluency in the SL, and attitudes toward CS. Furthermore, it responds to RQ5 by assessing the role of cultural heritage promotion, communication practices, and HL exposure in sustaining language transmission. Multilinear regression has been conducted to explore the predictors of HL transmission and maintenance. Due to the number of participants and the collinearity between the predictors, the independent variables have been selected based on the Pearson correlations. Due to the overlap between Personal and Environmental choices, only the first has been kept for the analysis. The predictor variables were the PCs obtained from the principal component analysis: the number of years in Israel, the SL Fluency (Köpke and Schmid, 2004), child-directed speech (Communication in the HL) and time spent with the child(ren) (Bedore et al., 2012; Gollan et al., 2015), and the personal choice of living in Israel. The findings are captured in Table 3. The predictors explained 55% of the total variance of parental views on HL transmission and maintenance. The findings indicate that parental attitude towards code-switching (*ß* = −3.131, *p* = 0.004) and transmission of cultural heritage (*ß* = 3.023, *p* = 0.006) are statistically significant predictors of HL maintenance.

**Table 3:** Summary of multilinear regression analyses for variables predicting HL maintenance.

Coefficients	Components		
	*ß*	*t*	*p*
Number of years in IL	0.081	−0.603	0.552
Personal choice	−0.176	−1.236	0.228
SL fluency	0.244	1.713	0.099
CS child	−0.436	−3.131	0.004*
Cultural heritage	0.466	3.023	0.006*

*Significant at the <0.001 level.

### 3.5 The underlying force of heritage language transmission and maintenance

To explore the role of pragmatic considerations in HL transmission and maintenance, this section addresses RQ6 and examines how intergenerational communication and literacy development shape parental motivations for HL preservation. There is a significant correlation between the parental efforts toward the importance of transmitting and maintaining the HL and the HL use by the children with family member living in the heritage country (*r_s_* = 0.884, *p* = 0.01). With regards to literacy skills, the data showed a significant correlation between the importance of HL knowledge and teaching literacy in the home environment (*r_s_* = 0.651, *p* < 0.001). Based on responses to the open-ended questions in the questionnaire, the TA (Braun and Cline, 2009) revealed that most participants held a traditional skill-based view of literacy, emphasizing the importance of mastering reading and writing. It also showed that they viewed literacy as essential for building vocabulary and achieving academic success.

*“It is important to me that s/he reads in Hungarian, as it develops vocabulary.”*

*(P18: “Fontos számomra, hogy könyvélményei is legyenek magyarul, és az olvasás ráadásul fejleszti a szókincset.”)*

Additionally, supporting multilingual literacy skills was also a key theme presented among the participants:

*“They learn English; therefore, they do not have problems with the Latin letters.”*

*(P23: “Úgyis tanulnak angolul, legalább nincsen gondjuk a latin abc-vel.”)*

Ultimately, the driving force of grandparents is also revealed in the parental view of literacy skills in the open-ended questions:

*“I find it important because of the family background.”*

*(P35: “Fontosnak tartom a családi háttér miatt.”)*

*“Because this is how they can talk to the grandparents.”*

*(P24: “Mert csak igy tudnak kommunikálni a nagyszülőkkel.”)*

The main findings of the parental self-reports indicated strong parental awareness and pragmatic reasons behind the necessity of bilingual education, especially in the HL at home.

## 4 Discussion

The HL maintenance among Hungarian-speaking immigrants in Israel is influenced by cultural identity and parental attitudes with a shift from assimilation to HL preservation due to the value of multilingualism in their education in post-communist Hungary. While family cohesion, especially the role of grandparents, supports HL transmission and maintenance, many parents invest less time than ideal, and their negative attitude towards CS reflects a preference for linguistic purity. However, the study’s findings are limited to a highly educated, mid-to-high SES sample. The results for each research question are presented and analyzed individually.

### 4.1 Family language policies and identity factors (RQ1 and RQ2)

FLP and identity are deeply interconnected, as FLP reflects parents’ linguistic choices, beliefs, and practices shaped by their cultural and personal identities. In transnational families, language use at home is not only a practical decision but also involves negotiating belonging, heritage, and integration into the host society. By examining FLP and identity together, this section highlights how participants’ family language practices mirror their evolving identities, balancing assimilation into Israeli society with maintaining their HL.

When people move from one country to another, their cross-cultural, emotional, and behavioral boundaries change, and they find themselves bridging these societies. The study participants differ from previous waves of Hungarian immigrants in several ways. Their decision was not led by traumatic historic events, nor economic reasons, but rather personal reasons. However, they show a shifting tendency between the former and recent immigrants, moving from assimilation towards preservation of the HL. The participants’ socialization took place after the fall of the communist regime while the home country was adopting European principles and became a member state of the EU. Therefore, it is assumed that it directed the participants to look at multilingualism as an asset, as it is given to children by birthright. This assumption is supported by the PCA results, which revealed that participants’ motivations for immigration were primarily personal rather than economic or political (KMO = 0.703, *p* < 0.001). The results are also aligned with previous studies as most participants are highly educated and exhibit positive attitudes towards HL transmission and maintenance (Guardado and Becker, 2014; Zhang and Slaughter-Defoe, 2009), and they embraced pro-bilingual and multilingual FLP. This is further reflected in the findings, which show that most families use HL and SL equally at home (Graph 1). While about half of the population speaks more than one language in their households (Meir et al., 2021, pp. 131–133), parents seem not to be aware of it. Rather, the results seem to highlight the tension between the assimilation to the host society and maintaining the HL within the transnational family. The emergence of distinct dimensions related to personal choice and environmental factors underscores the interplay between individual identity and sociocultural context in language maintenance. As mostly personal reasons drove the participants’ immigration, a transitional identity emerges as their choice of living in Israel is motivated by conscious decisions (Personal Choice component of the PCA) parallel to an unicultural identity (Environmental Reasons component of PCA). The importance of SL emerges not just due to its position and the majority language but also as a symbol of nation and unity. This is supported by the SL Fluency PCA results, where participants reported high motivation to acquire Hebrew. These bilingual families, reflecting both personal and environmental identities, were able to maintain fluency in Hebrew while also preserving their HL.

### 4.2 Parental attitudes and family efforts in heritage language transmission (RQ3)

Embracing the cultural heritage and its interrelation with HL transmission and maintenance shows that the participants recognize the contribution of cultural identity to HL maintenance. This finding resonates with studies highlighting the role of parental attitudes and cultural identity in language maintenance (Wei, 2009) and the relationship between socio-emotional outcomes and FLP, as it involves general well-being (King and Fogle, 2008; Okita, 2002; Tannenbaum and Howie, 2002), identity (Soehl, 2016) and family relations (De Houwer, 2007; Okita, 2002).

The phenomenon of HL management being demanding and effortful has emerged in the frequency data showing the mismatch between the highly rated importance of HL transmission and maintenance and the quantity of time parents invest in HL (33% of the participants spend less time than the recommended). The multilinear regression analysis revealed that parental attitudes towards CS and transmission of cultural heritage significantly predict HL maintenance. The negative attitude towards code-switching suggests a preference for maintaining linguistic boundaries within the HL environment, aligning with research emphasizing how some families restrict CS to reinforce HL use within the home (Grosjean, 2008).

### 4.3 Predictors of heritage language maintenance (RQ4)

The emphasis on transmitting cultural heritage also reflects parents’ pragmatic considerations and desire to preserve linguistic and cultural ties with their heritage country (Wei, 2018). Regression analysis results confirmed that cultural heritage transmission is one of the strongest predictors of HL maintenance, highlighting its central role in shaping parental efforts.

The data also indicate that parental attitudes towards codeswitching and cultural heritage are significant predictors of HL transmission and maintenance; they also emphasize parental roles in creating needs and opportunities for HL maintenance (e.g., Guardado and Becker, 2014; King and Fogle, 2008; Nesteruk, 2010; Zhang and Slaughter-Defoe, 2009).

### 4.4 Role of cultural heritage and exposure in heritage language transmission (RQ5)

Parents also view literacy development in Hungarian as important for cultural reasons and for enriching their children’s vocabulary. TA results confirm this, as parents frequently expressed that literacy in Hungarian plays a role in vocabulary growth and academic success.

Bilingual education is seen as valuable, not only for communication but for maintaining family bonds and cultural identity. Positive outcomes have also been observed, as a broader sense of family cohesion involving relatives, particularly grandparents in the heritage country, and its effects on a strong emotional connection to the HL were evident in the parental responses, where 90% of families reported having extended family in Hungary. Grandparents may serve as agents for HL transmission and maintenance because family members look at them as a value (Nguyen et al., 2023; Schwartz, 2008). The relationship between the strong ties with grandparents and HL maintenance is likely reciprocal, making it difficult to determine which one primarily drives the other. It is unclear whether strong family bonds encourage greater HL use or if active HL maintenance strengthens family cohesion. Family cohesion can cause a more significant effort to maintain the HL, and a greater effort to maintain the HL can positively influence the cohesion of transnational families.

### 4.5 Pragmatic considerations and intergenerational dynamics (RQ6)

Israel’s linguistic diversity, shaped by waves of immigration, reflects the richness and the challenges of maintaining multiple languages in a national context. Languages hold different levels of prestige based on their global influence (Bourdieu, 1991; De Swaan, 2001), with some languages (e.g., English, French or Russian) enjoying higher status while others (Polish, Hungarian, Welsh, etc.) may be perceived as less prestigious. Despite the vital cultural and identity-related role of the HL in an immigrant community, societal pressure often favors the SL for integration. Therefore, HLs with lower prestige are not always valued. However, the members of the extended transnational families (e.g., grandparents remained in the heritage country) play a crucial role in HL transmission, driven by both cultural commitment and pragmatic considerations. This is reinforced by the strong correlation between parental efforts to maintain HL and its use in communication with family members abroad, highlighting the role of intergenerational ties.

## 5 Conclusion

While much of the existing research on HL focuses on large and cohesive immigrant groups (e.g., Spanish or Russian), this study provides novel insights into how HL transmission, maintenance, and FLP unfold in a small and underrepresented immigrant community (Hungarian-speakers) embedded in a multilingual society, yet one that promotes using SL in all domains of everyday life (Israel). The study adopts a mixed-methods approach, combining quantitative analyses (PCA, regression) with qualitative analysis (TA). Hungarian is a low-prestige HL in Israel compared to higher-status immigrant languages like Russian or English. Yet, the study shows that many parents actively work to maintain Hungarian, suggesting that language prestige is not the sole factor in HL maintenance. While it is often assumed that families who exclusively prioritize HL would be the strongest supporters of its maintenance, the study’s unique contribution lies in the observation that families who find a balance between HL and SL exposure tend to sustain HL more effectively than strictly HL-focused families. It also highlights the critical role of intergenerational ties, demonstrating how family networks, particularly grandparents, contribute to linguistic and cultural continuity. By exploring how parental attitudes and cultural identity shape HL maintenance, the findings emphasize the active and dynamic process that requires joint and extended family effort rather than an automatic outcome of bilingual upbringing.

The study was restricted regarding the selection of participants (highly educated recent immigrants and mostly mothers who belonged to mid-high SES). Therefore, the results can not be generalized to the entire Hungarian-speaking community in Israel. The rich dataset obtained in this study and the multiple RQs and variables could benefit from further analyses which were beyond the scope of the present paper.

Future studies could include lower-SES Hungarian-speaking families to help assess whether economic constraints and access to educational resources affect HL maintenance, addressing the sample limitations of this study. Future research could also benefit from direct observational methods or incorporating additional sources, such as teacher evaluations or recorded naturalistic speech samples. To address this point, in our future research, we aim to conduct sociolinguistic interviews with HSs in the presence of their children. The interviews will be coded to examine actual linguistic behavior and attitudes. Longitudinal research tracking HL transmission over multiple years could further help determine whether FLP strategies remain stable or change as children grow older and how they affect HL retention. Finally, cross-cultural comparisons between Hungarian-speaking immigrants in Israel and those in other countries, such as Canada, the U.S., or Australia, could highlight the role of different sociolinguistic environments in shaping HL transmission. By addressing these gaps in the study of small transnational migrant communities, future studies can contribute to a more nuanced and holistic perspective on HL maintenance, helping policymakers, educators, and immigrant families develop effective strategies for HL transmission and maintenance across generations.

## Data Availability

The raw data supporting the conclusions of this article will be made available by the authors, without undue reservation.
